# Cost effectiveness analysis of a polygenic risk tailored breast cancer screening programme in Singapore

**DOI:** 10.1186/s12913-021-06396-2

**Published:** 2021-04-23

**Authors:** Jerry Zeng Yang Wong, Jia Hui Chai, Yen Shing Yeoh, Nur Khaliesah Mohamed Riza, Jenny Liu, Yik-Ying Teo, Hwee Lin Wee, Mikael Hartman

**Affiliations:** 1 Life Sciences Institute, National University of Singapore, Singapore, Singapore; 2 Saw Swee Hock School of Public Health, National University of Singapore and National University Health Systems Singapore, Singapore, Singapore; 3 Department of Pharmacy, Faculty of Science, National University of Singapore, Singapore, Singapore; 4 Department of Surgery, Yong Loo Lin School of Medicine, National University of Singapore and National University Health Systems, Singapore, Singapore

**Keywords:** Breast cancer screening, Cost effectiveness analysis, Economic evaluation, Polygenic risk scores, Risk stratification

## Abstract

**Background:**

This study aimed to evaluate the cost-effectiveness of a breast cancer screening programme that incorporates genetic testing using breast cancer associated single nucleotide polymorphisms (SNPs), against the current biennial mammogram-only screening programme to aid in its implementation into the current programme in Singapore.

**Methods:**

A Markov model was used to compare the costs and health outcomes of the current screening programme, against a polygenic risk-tailored screening programme, which can advise a long-term screening strategy depending on the individual’s polygenic risk. The model took the perspective of the healthcare system, with a time horizon of 40 years, following women from the age of 35 to 74. Epidemiological and cost data were taken from Asian studies, and an annual discount rate of 3% was used. The model outcome was the incremental cost-effectiveness ratio (ICER), calculated from the difference in costs per quality-adjusted life year (QALY). Scenarios with varying risk thresholds for each polygenic risk group were examined. One-way and probabilistic sensitivity analyses were performed to assess parameter uncertainty.

**Results:**

The ICER for a polygenic risk-tailored breast cancer screening programme, compared with the current biennial mammogram-only screening programme, was − 3713.80 SGD/QALY, with incremental costs < 0 and incremental effects > 0. The scenario analysis of different polygenic risk cutoffs showed that the ICERs remain negative, with all ICERs falling within the south-east quadrant of the cost-effectiveness plane, indicating that tailored screening is more cost effective than mammogram-only screening, with lower costs and higher QALYs to be gained. This suggests that a polygenic risk-tailored breast cancer screening programme is cost effective, entailing lower cost than the current mammogram-only programme, while causing no additional harm to women.

**Conclusion:**

Results from this cost-effectiveness analysis show that polygenic risk-tailored screening is cost effective with an ICER of − 3713.80 SGD/QALY. Tailored screening remains cost effective even across varying percentile cutoffs for each risk group. While the results look promising for incorporating polygenic risk into the current breast cancer screening programme, further studies should be conducted to address various limitations.

**Supplementary Information:**

The online version contains supplementary material available at 10.1186/s12913-021-06396-2.

## Background

Breast cancer is the most prevalent cancer among women globally. In 2018 alone, approximately two million new cases of breast cancer were diagnosed, accounting for 11.6% of all cancers, resulting in more than 626,000 deaths [1]. As with the global trend, breast cancer is also the most common cancer among women in Singapore, accounting for 29.1% of all cancer diagnoses. The incidence rate of breast cancer has been steadily increasing and has almost tripled from 24.6 per 100,000 person-years in 1976 to 65.3 per 100,000 person-years in 2011–2015 [2]. In the 5-year period of 2011–2015, a total of 9634 new cases of breast cancer were diagnosed. In comparison, there were 3136 new cases of breast cancer in 2018 alone [3]. Furthermore, breast cancer is consistently the cancer type with the highest number of fatalities, accounting for 2105 women in the period of 2011–2015 [2]. The survival rates of women diagnosed with stage IV breast cancer are much lower compared to the survival rates of those diagnosed at the earlier stages. Hence, it is imperative that a screening programme considers all these factors and screens for breast cancer promptly and effectively.

Given the critical importance of early detection in improving breast cancer outcome in Singapore, the BreastScreen Singapore programme was established in 2002 by the Health Promotion Board and has promoted the early detection of breast cancer to improve mortality rates [4, 5]. Singapore adopts an age-based screening approach, where women aged 50 years or older are advised to go for a mammogram once every 2 years. While national mammography screening programmes have been widely implemented and shown to be cost effective in countries including the US [6], Australia [7], and South Korea [8], their respective guidelines for screening still vary. The current strategy in Singapore has its limitations, such as poor attendance, with only 66% of women aged 50 to 69 ever getting a mammogram in 2018; as well as high false positive rates and missed cases [9, 10].

Clearly, improvements can be made to the Singaporean age-based mammogram screening programme. Polygenic risk scores (PRS) have been shown to predict an individual’s risk of diseases [11–13]. Using breast cancer associated SNPs, an individual’s PRS can be calculated to stratify their risk of developing breast cancer, allowing for personalized recommendations for a significant portion of the population [14–16].. However, despite growing evidence of PRSes being a useful supplement to national screening programs, there has not been widespread implementation among countries that carry out age-based screening approaches [17]. At this stage, studies have been focusing on evaluating the performance of PRSes in a clinical setting. Most notably, cost-effectiveness analyses have been performed to assess the monetary and health benefits of incorporating PRSes in age-based population screening programmes. Many of these support a risk-based approach over current methods based on its cost-effectiveness and reduction of overdiagnosis, while retaining screening benefits [15, 18–20]. Large-scale trials such as WISDOM [21] and PROCAS [22] have been commenced in order to determine the impact of PRSes in population screening approaches. These would form the foundations for the informed implementation of PRSes in national age-based screening programs by policy makers [23, 24].

Hence, we recognize the potential of a genetic risk approach to breast cancer screening, and through this study, we will present a cost-effectiveness analysis on a genetic risk-based screening programme in the context of Singapore. We will introduce a polygenic risk-tailored screening programme which aims to facilitate early detection by providing tailored screening recommendations based on an individual’s risk group. This may reduce unnecessary testing and false positives that are common in a one size fits all mammogram screening programme, while also acting as a platform to increase awareness in personal breast cancer risks. We compare the current age-based biennial mammogram screening programme with a strategy that incorporates genetic testing using breast cancer associated SNPs, thus evaluating the cost-effectiveness of the genetic risk prediction approach for breast cancer in Singapore to aid in its implementation within the current strategy.

## Methods

**Fig. 1:**
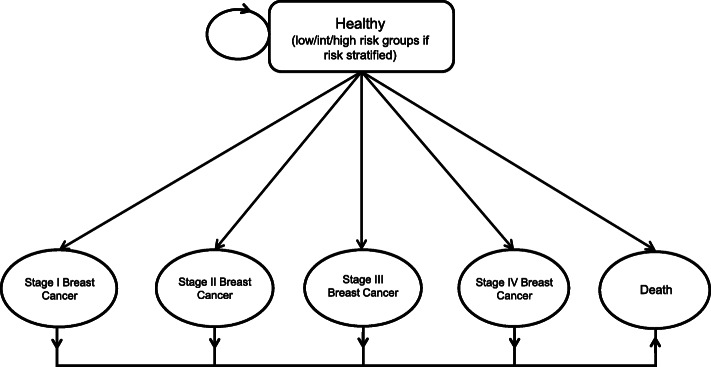
Markov Model of breast cancer progression. Patients diagnosed with breast cancer will transition into the stage-specific diseased states and remain there, as remission and treatment were not modeled. Patients who are healthy remain in a healthy state until diagnosis or death

**Fig. 2:**
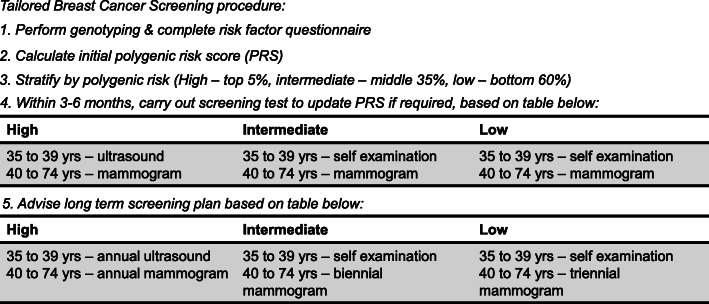
Summary of proposed polygenic risk tailored screening programme

The target population of this cost-effectiveness analysis is Singaporean women aged 35 to 74, a time horizon of 40 years. The two strategies being compared are – the proposed tailored screening strategy, which advises a screening programme based on an individual’s PRS, and the current mammogram screening only strategy, where mammogram screening is done every 2 years. A Markov model (Figs. 1 and 2) for breast cancer screening in Singaporean women was developed in Microsoft Excel to model the differences between the two strategies. This study takes the perspective of the healthcare system in Singapore.

In the polygenic risk tailored screening strategy, individuals aged 35 to 74 will be genotyped by buccal swab and asked to complete a questionnaire on breast cancer risk factors, before being stratified into three risk groups based on their initial PRS – low, intermediate, and high. The PRSs are stratified by setting cutoffs at below 60th percentile for the low-risk group, 60th to 95th percentile for the intermediate-risk group and above 95th percentile for the high-risk group. In the Asian population, these cutoffs correspond to expected proportions of 51, 41 and 8% for low-, intermediate- and high-risk groups respectively [study team unpublished data]. The starting age of 35 for this strategy was chosen based on the findings that the risk for women with a high genetic risk for developing breast cancer may be as high as the 10-year risk for an average 50-year old woman, already at 35 years of age [25]. There is no clear guideline on the ending age for mammogram. The U.S. Preventive Services Task Force (USPSTF) recommends screening up to the age of 74 [26]. The American Cancer Society recommends that mammogram may be performed as long as an individual is in good health and expected to live for another 10 years [27]. Hence, we have chosen an ending age of 74 for the tailored screening arm, to compare against the current Singapore mammogram-only strategy that screens every 2 years from the age of 50 to 69.

Individuals in each risk group will receive their initial PRSs within three to 6 months a buccal swab. Depending on their age group, these individuals will be required to undergo a subsequent follow-up screening test comprising of self-examination, ultrasound or mammogram (Fig. 1). Results from the follow-up screening tests will be used to give a holistic genetic score. Individuals will be advised with a long-term screening plan tailored to their updated risk group from this genetic score (see Fig. 1). In terms of age for the last screening, in the proposed tailored screening strategy, it is set at age 73 for the low-risk group and 74 for the intermediate- and high-risk groups, while for the mammogram arm it is at age 68, with no screening after the age of 69.

Parameters were extracted from Singaporean studies or closely related Asian studies if Singaporean equivalents were not available. Age-specific incidence rates (ASIR) for breast cancer were provided by the Demographic Epidemiological Model of Singapore (DEMOS), a published local micro-simulation disease model that synthesizes evidence from multiple data sources [28]. Screened and unscreened breast cancer stage distributions were taken from a previous study by Wong [29], where the MISCAN-Fadia model [30] was calibrated to Singaporean breast cancer incidence data. Mammography sensitivity was incorporated to account for any missed cases. To illustrate the differences between breast cancer incidence in each polygenic risk group, multipliers of 2x, 1x, and 0.5x were used for high-, intermediate-, and low-risk groups, respectively. Transition probabilities between healthy and each disease state were then calculated using these parameters (ASIR x Stage Specific Proportion x Mammography Sensitivity x Risk Multiplier (if applicable)). Mortality rates were derived from the Singapore Cancer Registry Annual Registry Report 2015 [2]. Direct medical costs by breast cancer stage and genotyping buccal swab cost were obtained from Wong [29]. The costs of mammogram and ultrasound tests were adapted from local public and private hospitals. All costs were expressed in Singapore Dollars (SGD). The health outcomes used were Life Years (LYs) gained and Quality-Adjusted Life Years (QALYs) gained. QALYs are calculated by multiplying the time spent in each health state with an appropriate utility score. We used the stage-specific health utility scores from Wong [29], which were adapted from a Korean study [31]. The discount rate used for the costs and health outcomes is 3% [32].

The attendance rate of 100% of all women was assumed. The model also assumes that all women can die from natural causes in between screening cycles. Those who are diagnosed with breast cancer do not go into remission and instead remain in the diseased states. Risk group multipliers were selected based on the assumption that high-risk women for instance, are approximately twice as likely as the average population to develop breast cancer. Low-risk women, conversely, would be half as likely as the average population to develop breast cancer [16, 26]. These assumptions were based on various PRS studies, for example Mavaddat et al. [14], who found that compared with women in the middle quintile of breast cancer PRSs, those in the top 1% had 4.37- and 2.78-fold risks of developing ER (Estrogen Receptor)-positive and ER-negative disease, respectively.

Probabilistic sensitivity analysis (PSA) and one-way sensitivity analysis (OSA) were carried out to assess parameter uncertainty. For OSA, the lower and upper limits of each parameter were calculated using 80 and 120% respectively of the original value. PSA was conducted on the cost parameters (+/− 30%, Gamma distribution) and utilities (+/− 0.1, Beta distribution). Ten thousand runs of Monte Carlo simulations were carried out for the PSA.

Given that the cut-offs are somewhat arbitrary, we evaluated the impact of adopting different cutoffs for the risk groups in a scenario analysis. For the high-risk group, cut-offs at 5th to 10th percentile was explored, while for the low-risk group, cut-offs at 40th to 60th percentile was explored. These ranges were covered by three separate scenarios – 1) 60th percentile low−30th percentile intermediate-10th percentile high (60 L-30I-10H), 2) 40th percentile low-55th percentile intermediate-5th percentile high (40 L-55I-5H), and 3) 40th percentile low-50th percentile intermediate-10th percentile high (40 L-50I-10H), all in comparison with the base-case scenario of 60th percentile low-35th percentile intermediate-5th percentile high (60 L-35I-5H) (Table 1).

**Table 1:** Input parameters for the cost-effectiveness model

Variables	Baseline	Minimum	Maximum	Distribution	Reference
Age Specific Incidence Rates		–	–	–	Demographic Epidemiological Model of Singapore, DEMOS [28]
*35–39*	0.000617				
*40–44*	0.001114				
*45–49*	0.001733				
*50–54*	0.001775				
*55–59*	0.002073				
*60–64*	0.002119				
*65–69*	0.002056				
*70–74*	0.002063				
*75–79*	0.001974				
*80–84*	0.001710				
*> = 85*	0.001530				
Annual discount rate for costs and benefits	0.03	–	–	–	Haackeret al. (2020) [32]
Stage Specific Mortality Rates		–	–	–	Singapore Cancer Registry Annual Registry Report 2015 [2]
*Stage I*	0.020				
*Stage II*	0.044				
*Stage III*	0.083				
*Stage IV*	0.268				
All-Cause Mortality Rate	0.002896	–	–	–	Singstat Life Tables [33]
Polygenic Risk Distribution		–	–	–	Study team’s unpublished data
*Low*	0.51				
*Intermediate*	0.41				
*High*	0.08				
Breast Cancer Stage Distribution (Screened/Unscreened)		–	–	–	Wong (2019) [29]
*Stage I*	0.53/0.22				
*Stage II*	0.43/0.57				
*Stage III*	0.03/0.12				
*Stage IV*	0.01/0.09				
Stage Specific Utility Values				Beta	Wong (2019) [29]
*Healthy*	1.000	1.000	1.000		
*Stage I*	0.731	0.63	0.83		
*Stage II*	0.731	0.63	0.63		
*Stage III*	0.599	0.499	0.599		
*Stage IV*	0.352	0.252	0.452		
Risk Group Multiplier		–	–	–	–
*High*	2				
*Intermediate*	1				
*Low*	0.5				
Mammogram & Ultrasound Sensitivity	0.8	–	–	–	[27–30]
Stage Specific Direct Medical Costs (SGD)				Gamma	Wong (2019) [29]
*Stage I*	63,983.00	44,788.10	83,177.90		
*Stage II*	78,226.00	54,758.20	101,693.80		
*Stage III*	91,129.00	63,790.30	118,467.70		
*Stage IV*	110,136.00	77,095.20	143,175.80		
Cost of Buccal Swab (SGD)	210.00	122.50	227.50	Gamma	Local Singapore Hospitals, Wong (2019) [29]
Cost of Mammogram (SGD)	110.00	87.50	162.50	Gamma	Local Singapore Hospitals, Wong (2018) [20], Wong (2019) [29]
Cost of Ultrasound (SGD)	230.00	161.00	299.00	Gamma	Local Singapore hospitals, Wong (2019) [29]
Cost of Questionnaire (SGD)	2.00	1.40	2.60	Gamma	Sun et al. (2018) [19]

## Results

The cost-effectiveness model estimated 25.5 cases of breast cancer per 1000 women over the time horizon of ages 35–74 years old in the tailored screening arm, compared to 31.2 cases in the mammogram screening arm. Overall, the life year and quality-adjusted life year gain per woman in the tailored screening programme is approximately 0.9720 and 0.9884, respectively. The tailored screening programme is cheaper by SGD3,670.83, resulting in an ICER of − 3713.80 SGD/QALY (Table 2).

**Table 2:** Costs and health outcomes of the cost effectiveness model for two screening strategies

***Strategy***	Lifetime costs per case (SGD)	Life Years	Quality- Adjusted Life Years (QALYs)	Incremental Calculations (Tailored – Current)			
				Costs	Life Years	QALYs	ICER (SGD/QALY)
Current mammogram only screening	23,729.57	21.89	21.80	–	–	–	–
Polygenic Risk Tailored Screening (60th low, 35th int, 5th high)	20,058.74	22.86	22.79	-3670.83	0.9720	0.9884	-3713.80
*Scenario Analysis*							
60th low, 30th int, 10th high	21,474.94	22.86	22.78	-2254.63	0.9686	0.9800	-2300.45
40th low, 55th int, 5th high	22,242.34	22.85	22.77	-1487.23	0.9599	0.9681	-1536.20
40th low, 50th int, 10th high	23,658.54	22.85	22.76	−71.02	0.9566	0.9598	−74.00

Summary of cost and health outcomes of the cost-effectiveness analysis, in terms of costs, life years, quality-adjusted life years (QALYs), and the incremental cost-effectiveness ratio (ICER). The strategies being compared are the current mammogram-only screening strategy and a proposed polygenic risk-tailored screening strategy. The base polygenic risk-tailored screening strategy was cheaper by SGD -3670.83, with a QALY gain per woman of 0.9884, giving a negative ICER of −3713,80 SGD/QALY over the age-based mammogram-only programme. Scenario analysis compared three scenarios (60 L-30I-10H, 40 L-55I-05H, 40 L-50I-10H) with different polygenic risk cutoffs in percentiles for polygenic risk tailored screening, and for all three scenarios, the ICER was negative (−2300.45 SGD/QALY, −1536.20 SGD/QALY, − 74.00 SGD/QALY)

Three scenarios with different percentile cutoffs were explored with splits of 60 L-30I-10H, 40 L-55I-5H, 40 L-50I-10H, giving ICERs of − 2300, − 1536, − 74 SGD/QALY, respectively. The ICERs for all three scenarios were negative and remained in the south-east quadrant of the cost-effectiveness plane with a negative incremental cost and positive incremental QALYs.

**Fig. 3:**
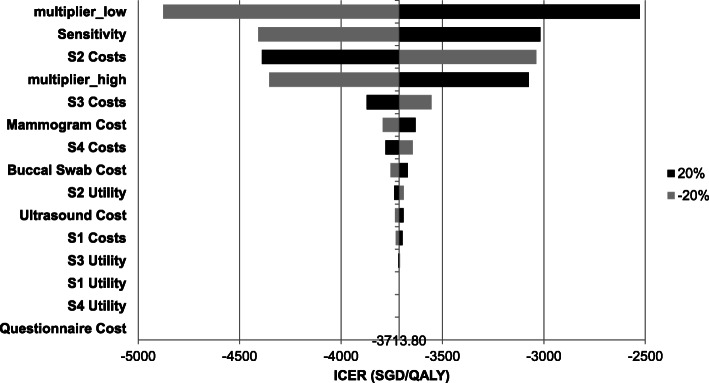
Tornado diagram of one-way sensitivity analysis

To assess the impact of the parameters on the health outcomes and ICERs, both one-way and probabilistic sensitivity analyses were conducted. Figure 3 shows the tornado diagram for the OSA. Low- and high-risk multipliers, direct medical costs for Stage II breast cancer, and sensitivity of mammogram and ultrasound tests were the top four parameters that most affected the ICER. Nevertheless, the ICERs remain negative, with incremental costs < 0 and incremental effects > 0.

**Fig. 4:**
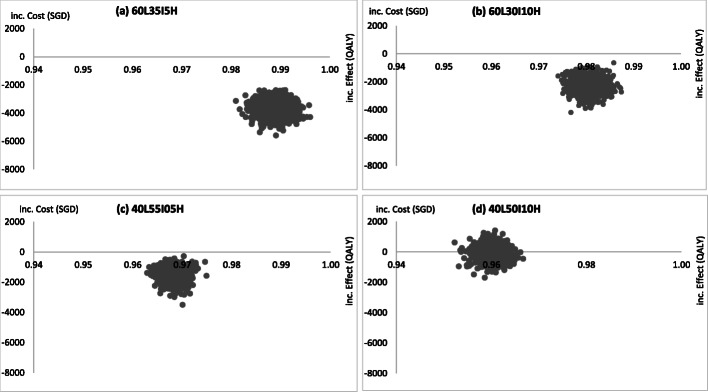
Cost effectiveness planes for probabilistic sensitivity analysis for all four scenarios in scenario analysis. Legend: In the 40 L-50I-10H scenario (**d**), approximately 57% of the ICERs for tailored screening will be cost effective when WTP is at 1SGD /QALY, compared to the mammogram arm. All ICER points for the three other scenarios ((**a**), (**b**), (**c**)) remain in the south-east quadrant, indicating that tailored screening is more cost effective than mammogram-only screening, entailing lower costs and higher QALY gain in these scenarios

**Fig. 5:**
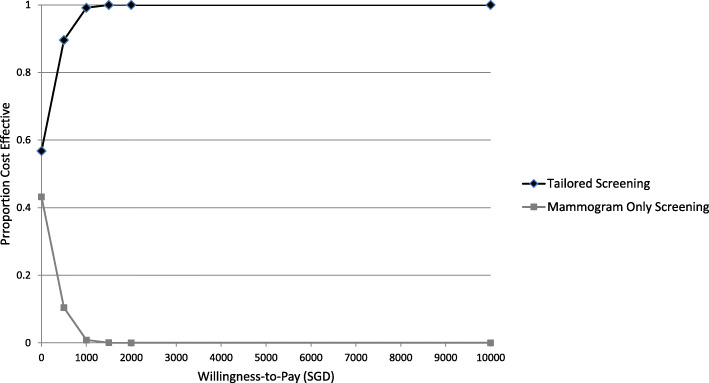
Cost effectiveness acceptability curve for the 40 L-50I-10H scenario

Figure 4a shows the cost-effectiveness plane from the PSA on the baseline scenario, where all ICER points fall within the south-east quadrant, indicating that tailored screening is more cost effective compared to mammogram-only screening, with lower costs and higher QALYs. After 10,000 runs of Monte Carlo simulations, the probability of tailored screening being more cost effective compared to the mammogram screening arm is 100%, with the ICER remaining negative with incremental costs of < 0 and incremental effects of > 0. The willingness-to-pay (WTP) threshold represents an estimate of what an individual is willing to pay for a health benefit, in this case for gaining 1 QALY. Among the PSA done on the three additional scenarios with different risk group cutoffs, of note is the 40 L-50I-10H scenario where the ICER crosses from negative to positive. Approximately 57% of the ICERs for tailored screening will be cost effective when WTP is at 1SGD /QALY, compared to the mammogram arm (Fig. 3). At maximum WTP of 1820 SGD/QALY, tailored screening is 100% cost effective. In other scenarios, tailored screening dominates mammogram-only screening (Fig. 5).

## Discussion

A breast cancer screening programme incorporating polygenic risk scores could be implemented in Singapore to improve the current age-based mammogram screening programme. The cost-effectiveness of genetic risk-tailored screening policies have been studied in the United Kingdom [15], Canada [34], and the United States [35]. However, little was known of the cost-effectiveness of such policies in Asia. Hence, we developed a cost-effectiveness model to examine the feasibility of a genetic risk-tailored screening approach in Singapore, where individuals would be advised on a screening strategy based on their predicted risk.

Our results from the cost-effectiveness analysis suggest that a polygenic risk-based tailored screening approach is cost effective over the current age-based mammogram-only screening programme in Singapore. An ICER of − 3713.80 SGD/QALY makes polygenic risk-based tailored screening more cost effective than the current mammogram screening programme. With the incremental cost of -SGD3,709, tailored screening is evidently less costly. This result is supported by a number of Western studies that indicate that a polygenic risk-stratification component may improve cost-effectiveness of a breast cancer screening programme [17, 34, 35]. In terms of ending age, we opted for the age 74 based on American recommendations, over mirroring the current strategy’s ending age of 69. This choice in comparison would give an ICER of − 3717.72 SGD/QALY, showing that the strategy remains cost-effective even with adjustments.

In terms of health outcomes, the observed small difference in QALYs of 0.9853 suggests that tailored screening does not differ considerably from mammogram-only screening in terms of QALYs gained, with almost one QALY gained per individual. This implies that tailored screening does not result in a significant shift in the stage distribution of breast cancer cases. We believe that this is a positive finding, as it indicates that no harm will be inflicted upon individuals by transitioning from mammogram-only screening to polygenic risk-tailored screening [36]. The findings further indicate no significant difference in cancer survival, as quality of life is tied to cancer staging and survival time. It was also observed that the probability of death over lifetime per individual does not differ significantly between the two arms - 13.0% in the tailored screening arm and 13.4% in the mammogram screening arm. This further demonstrates that there are no adverse outcomes to the patient when switching to the tailored screening programme.

Overall, a polygenic risk-tailored screening program proved to be more cost-effective compared with the current age-based mammogram-only strategy, offering both health and monetary advantages. This study would provide the framework and foundation for policy- and decision-makers to introduce genetic risk-based methods into the current screening program, especially in the context of Asia, as preceding cost-effectiveness analyses on risk-based screening methods have mainly been done in Western countries [18, 37]. This cost-effectiveness study would also pave the way for exploring other social and economic issues such as manpower, access and regulation to be addressed when implementing a risk-based approach in national breast cancer screening programmes [15, 17, 38]. By comparing outcomes using varying percentile cutoffs for the different risk groups, the study demonstrated that the tailored-screening strategy can be flexible, allowing policy makers to adjust strategies depending on local population data. Notably, the 40 L-55I-10H scenario, which is when stratification is weighted towards those at high risk, has an ICER of − 74.00 SGD/QALY. This is due to a shift in screening frequencies, with more screening done in the high-risk groups due to the higher proportions, resulting in higher costs, driving the ICER up. Nonetheless, at the approximate maximum WTP of 1820 SGD/QALY, tailored screening is 100% cost-effective. Our cost-effectiveness model estimated approximately 25.5 cases of breast cancer per 1000 women, over the time horizon of 35–74 years old in the tailored screening arm. In the mammogram-only screening arm, the model estimated 31.2 cases per 1000 women. Based on 2018 statistics from Globocan, women in Singapore have a cumulative risk of 6.39% of developing breast cancer, which equates to 60.9 cases per 1000 women. While this shows that the model may have underestimated the number of breast cancer cases, it has to be noted that the model only covers women aged 35–74, a subset of all women in Singapore.

This study used model-based estimates based on assumptions. The model assumes 100% attendance and compliance with breast cancer screening and follow-ups, which is not representative of the real-world situations. A 2010 national health survey (in Singapore) showed that only 39.6% of women aged 50–69 years old have attended screening in the previous 2 years [39]. In comparison, screening attendance according to national guidelines was 61.1% in South Korea in 2010 [40]. Mammography sensitivity was a limitation in this study, as scarcity and age of the data may impact the results. The Singapore MOH Clinical Practice Guidelines 2010 estimated the sensitivity of mammography to range from “68% to over 90%” [41]. Hence, we set our mammography sensitivity parameter to a base case value of 80%, varying it from 64 to 96% in the one-way sensitivity analysis. There is also a risk of confounding due to the use of observed screening outcomes rather than natural history data. However, this limitation applies to both intervention and control and the effects will be cancelled out as we are interested in marginal analysis. Another limitation of our study is that we did not model for treatment, remission and follow-ups after diagnosis. However, this should not affect the conclusion as we expect the number of breast cancers detected by screening to be a proxy for these longer-term outcomes.

We are aware that demonstrating cost-effectiveness is only the first step. There are many other barriers when it comes to implementing such a risk-based cancer screening programme, as we have seen in other examples [42]. Successful implementation requires the buy-in of relevant stakeholders which include decision makers, primary care and specialist physicians and screen-eligible women, appropriate funding mechanism, tried-and-tested workflow for the return of results, tracking of follow-ups and outcomes, infrastructure for doing genetic testing at scale and many other considerations. The translation from concept to reality is not trivial.

## Conclusions

We carried out a cost-effectiveness analysis comparing the current age-based mammogram breast cancer screening programme in Singapore, against a genetic risk-tailored programme. Our results show that tailored screening is cost-effective with an ICER of − 3713.80 SGD/QALY, while being less costly with no additional harm. Tailored screening remains cost-effective even when varying percentile cutoffs for each risk group. These results are crucial for policymakers in demonstrating the feasibility of a risk-based approach in Singapore. However, while the results may serve as important foundations for a risk-based approach to be implemented, further studies should be conducted to address the limitations related to data availability and modeling.

## Supplementary Information

**Additional file 1:** **Supplementary Figure 1.** Cost effectiveness acceptability curve for the baseline scenario (60L-35I-5H).

**Additional file 2: Supplementary Figure 2.** Cost effectiveness acceptability curve for the 60L-30I-10H scenario.

**Additional file 3: Supplementary Figure 3.** Cost effectiveness acceptability curve for the 40L-55I-5H scenario.

**Additional file 4.** Excel model file for the 40L-50I-10H scenario.

**Additional file 5.** Excel model file for the 40L-55I-5H scenario.

**Additional file 6.**Excel model file for the 60L-30I-10H scenario.

**Additional file 7.** Excel model file for the 60L-35I-5H scenario.

## Data Availability

The datasets used and/or analysed during the current study are available from the corresponding author on reasonable request.

## Abbreviations

SNPs: Single Nucleotide Polymorphisms
ICER: Incremental Cost-Effectiveness Ratio
LY: Life Year
QALY: Quality Adjusted Life Year
PRS: Polygenic risk score
SGD: Singaporean Dollars
ASIR: Age specific incidence rates
MISCAN-Fadia: MIcrosimulation SCreening Analysis (MISCAN) Fatal Diameter
ER: Estrogen receptor
PSA: Probabilistic sensitivity analysis
OSA: One-way sensitivity analysis
H: High risk
I: Intermediate risk
L: Low risk
WTP: Willingness-to-pay
MOH: Ministry of Health
DCIS: Ductal carcinoma in situ
