# Supplementary figures and images for: Cost effectiveness analysis of a polygenic risk tailored breast cancer screening programme in Singapore
Source: BMC Health Serv Res. 2021 Apr 23;21:379. doi: 10.1186/s12913-021-06396-2 (PMC8066868; doi:10.1186/s12913-021-06396-2)

## Slide 1
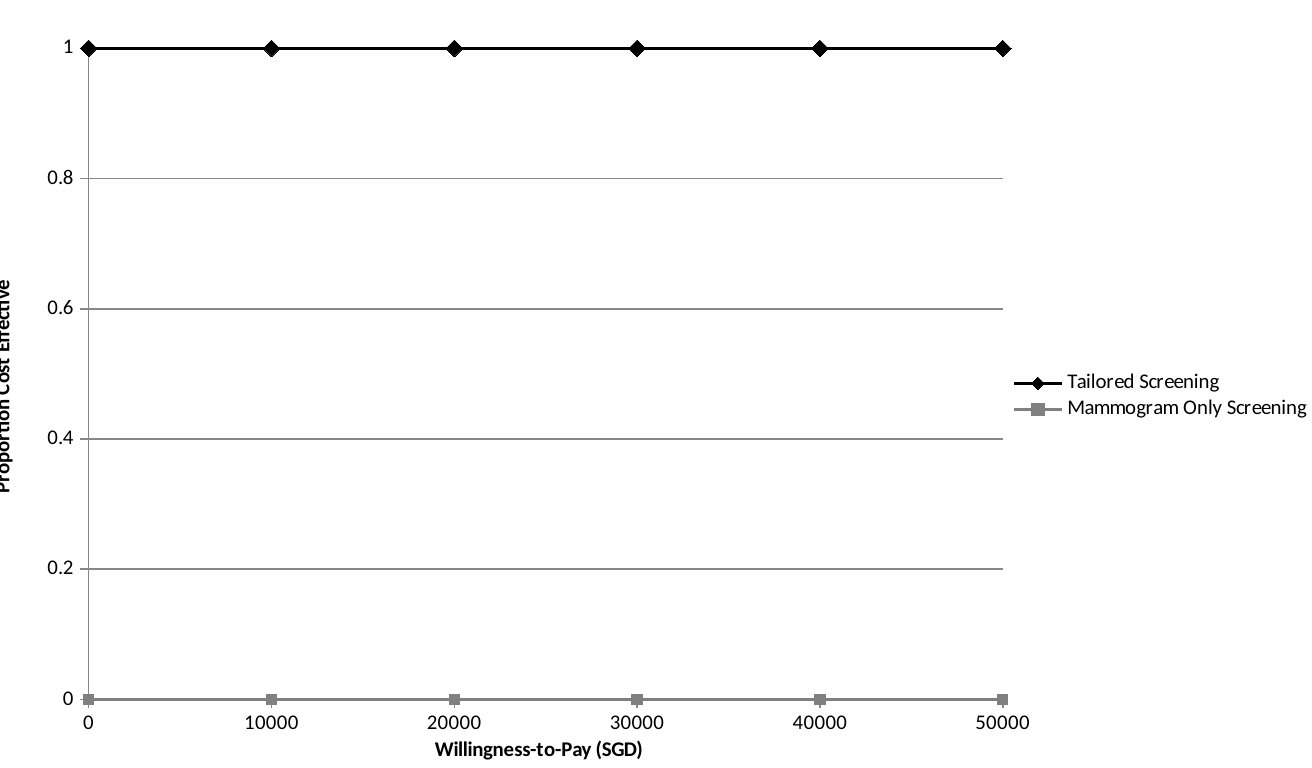

### Chart
| Category | Tailored Screening | Mammogram Only Screening |
|---|---|---|

Supplement: Supplementary file 1 — Additional file 1: Supplementary Figure 1. Cost effectiveness acceptability curve for the baseline scenario (60L-35I-5H). [file 12913_2021_6396_MOESM1_ESM.pptx]

## Slide 1
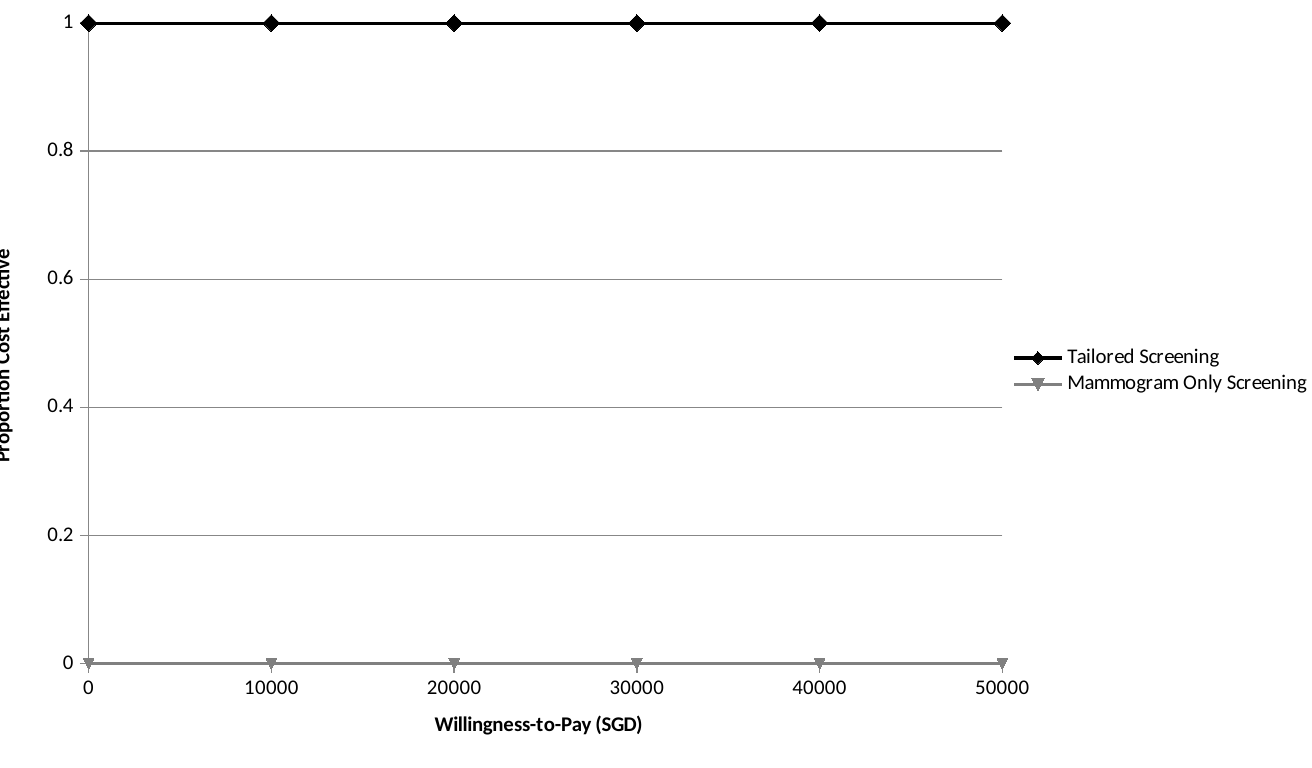

### Chart
| Category | Tailored Screening | Mammogram Only Screening |
|---|---|---|

Supplement: Supplementary file 2 — Additional file 2: Supplementary Figure 2. Cost effectiveness acceptability curve for the 60L-30I-10H scenario. [file 12913_2021_6396_MOESM2_ESM.pptx]

## Slide 1
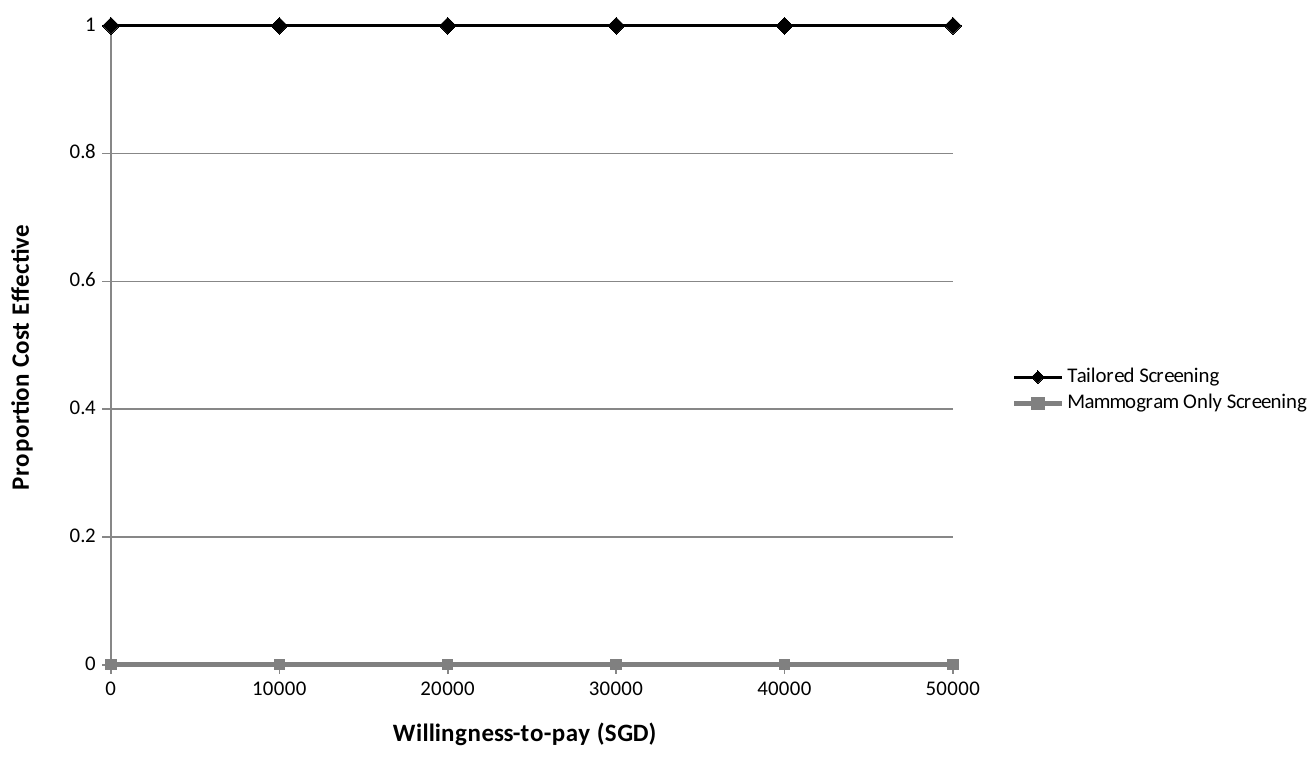

### Chart
| Category | Tailored Screening | Mammogram Only Screening |
|---|---|---|

Supplement: Supplementary file 3 — Additional file 3: Supplementary Figure 3. Cost effectiveness acceptability curve for the 40L-55I-5H scenario. [file 12913_2021_6396_MOESM3_ESM.pptx]
